# Intracellular toxic advanced glycation end-products in cardiomyocytes may cause cardiovascular disease

**DOI:** 10.1038/s41598-019-39202-5

**Published:** 2019-02-14

**Authors:** Takanobu Takata, Akiko Sakasai-Sakai, Tadashi Ueda, Masayoshi Takeuchi

**Affiliations:** Department of Advanced Medicine, Medical Research Institute, Kanazawa Medical University, Uchinada-machi, Ishikawa 920-0293 Japan

## Abstract

Cardiovascular disease (CVD) is a lifestyle-related disease (LSRD) and one of the largest public health issues. Risk factors for CVD correlate with an excessive intake of glucose and/or fructose, which has been shown to induce the production of advanced glycation end-products (AGEs). We previously identified AGEs derived from glyceraldehyde and named them toxic AGEs (TAGE) due to their cytotoxicities and relationship with LSRD. We also reported that extracellular TAGE in the vascular system may promote CVD and that serum TAGE levels are associated with risk factors for CVD. The mechanisms responsible for the onset and/or progression of CVD by extracellular TAGE or the above risk factors involve vascular disorders. In the present study, we revealed that rat primary cultured cardiomyocytes generated intracellular TAGE, which decreased beating rates and induced cell death. LC3-II/LC3-I, a factor of autophagy, also decreased. Although intracellular TAGE may be targets of degradation as cytotoxic proteins *via* autophagy, they may inhibit autophagy. Furthermore, the mechanisms by which intracellular TAGE decrease beating rates and induce cell death may involve the suppression of autophagy. The present results suggest that intracellular TAGE are generated in cardiomyocytes and directly damage them, resulting in CVD.

## Introduction

Cardiovascular disease (CVD) is a lifestyle-related disease (LSRD) and one of the largest public health issues of this century. Although CVD is associated with diabetes mellitus (DM)^1–5^, recent investigations revealed that the risk of CVD has increased in healthy humans due to a lifestyle that includes abundant amounts of calorie-rich food^6,7^. Relationships between an excessive intake of glucose and/or fructose and risk factors for CVD have been indicated not only in DM patients, but also in healthy humans^3–12^. Glucose and/or fructose have been shown to induce the production of advanced glycation end-products (AGEs)^12–20^, and toxic and non-toxic AGEs exist among the various types of AGE structures generated *in vivo*. We previously identified AGEs derived from glyceraldehyde (GA), the glucose and/or fructose metabolism intermediate, named toxic AGEs (TAGE) because of their cytotoxicities and involvement in LSRD, such as CVD, hypertension, insulin resistance, diabetic vascular complications, nonalcoholic steatohepatitis, Alzheimer’s disease, and cancer^13–20^.

We also reported that extracellular TAGE in the vascular system may promote CVD and that serum TAGE levels correlated with risk factors for CVD^1,13,14,20–28^. The mechanisms by which extracellular TAGE or the above risk factors promote CVD involve vascular disorders. However, it currently remains unclear whether intracellular TAGE are generated in cardiac cells. We hypothesized that intracellular TAGE may be generated in and damage cardiac cells because they were previously shown to be produced in neuroblastoma cells^16^, hepatic cells^29–31^, and pancreatic cells^32^ and induced cell death. If our hypothesis is correct, intracellular TAGE in cardiac cells may cause damage, resulting in CVD without vascular disorders. Therefore, we considered it important to investigate the generation of TAGE in cardiomyocytes because the beating rate is a very important and characteristic function of cardiac cells^33–36^.

In the present study, rat primary cultured cardiomyocytes were treated with GA. Beating rates, cell viability, and the generation of intracellular TAGE were analyzed. We also investigated whether cardiomyocytes degrade intracellular TAGE as long-lived, aggregated, or misfolded proteins *via* autophagy^37–39^. To achieve this, we analyzed LC3-II/LC3-I and p62, which are factors of autophagy.

## Results

### Beating rates of cardiomyocytes treated with GA

**Figure 1:**
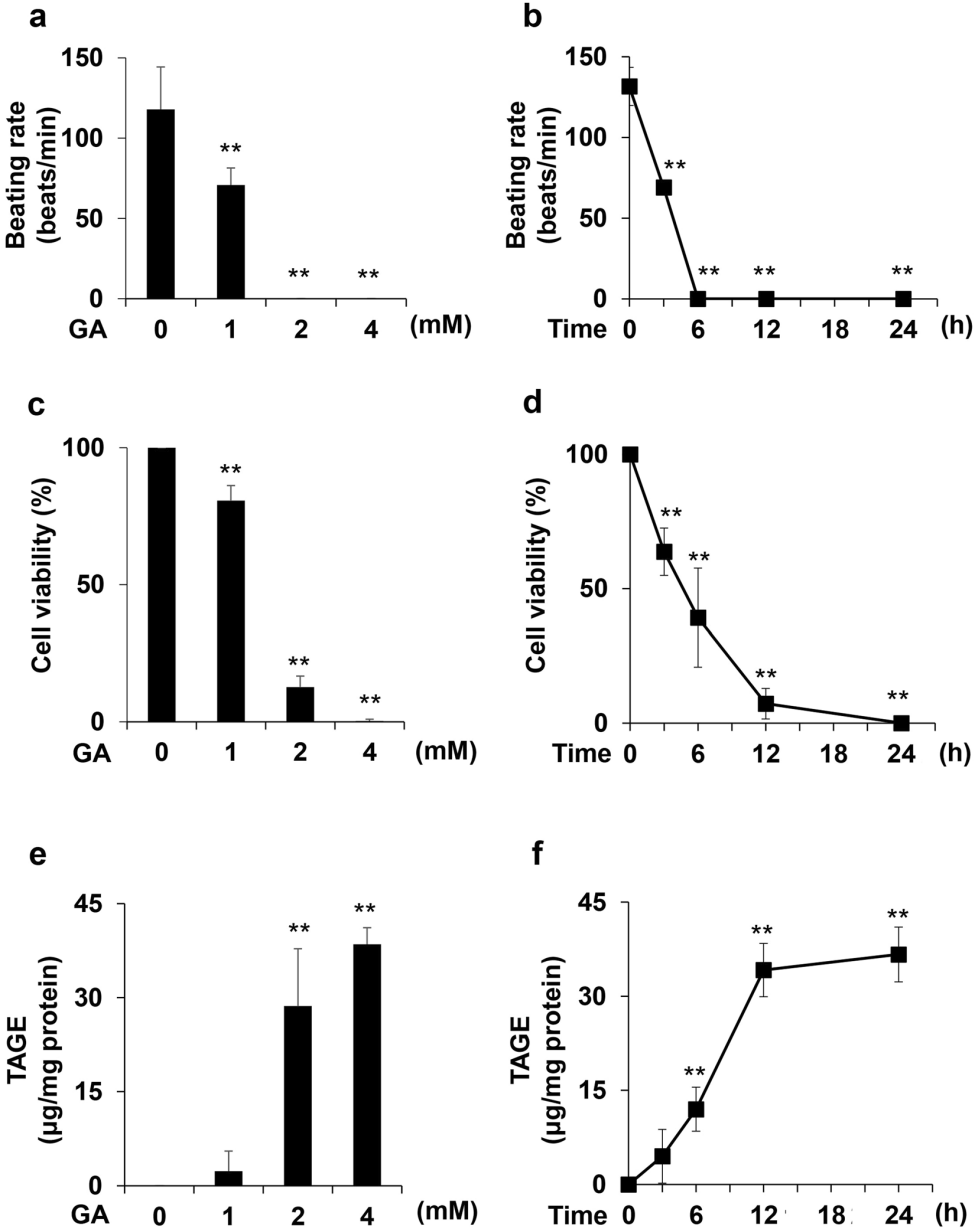
The beating rate, cell viability, and quantity of intracellular TAGE in cardiomyocytes treated with GA. (**a**,**b**) Beating rates were assessed in three independent experiments. One experiment was performed to count the beating rates of cardiomyocytes in 4 circular areas (diameter of 2 mm) in 35-mm dishes in order to calculate the average. Data are shown as means ± S.D. (N = 3). P-values were based on Dunnett’s test. **p < 0.01 vs. the control. (**c**,**d**) Cell viability was assessed by the WST-8 assay. This assay was performed in three independent experiments. One experiment was performed using 4 wells to calculate the average. Data are shown as means ± S.D. (N = 3). P-values were based on Dunnett’s test. **p < 0.01 vs. control. (**e**,**f**) Intracellular TAGE were analyzed with a slot blot (SB) analysis. Cell lysates (2.0 μg of protein/lane) were blotted onto a polyvinylidene difluoride (PVDF) membrane. The amount of TAGE was calculated based on a calibration curve for GA-derived AGE-BSA (TAGE-BSA). A SB analysis was performed in three independent experiments. Data are shown as means ± S.D. (N = 3). P-values were based on Dunnett’s test. **p < 0.01 vs. the control.

The beating rates of cardiomyocytes treated with 0, 1, 2, and 4 mM GA for 24 h decreased in a dose-dependent manner (Fig. 1a), while the beating of cardiomyocytes treated with 2 and 4 mM GA for 24 h completely stopped. The beating rates of cardiomyocytes treated with 4 mM GA for 0, 3, 6, 12, and 24 h markedly decreased in a time-dependent manner (Fig. 1b). The beating rate of cardiomyocytes incubated for 3 h was 69 beats/min, compared with 132 beats/min at 0 h. The beating of cardiomyocytes completely stopped 6 h after the GA treatment, and the cessation of beating was maintained for 12 and 24 h.

### Cell viability of cardiomyocytes treated with GA

The cell viability of cardiomyocytes treated with 0, 1, 2, and 4 mM GA for 24 h was assessed using the WST-8 assay. Cell viability decreased in a dose-dependent manner (Fig. 1c). The cell viability of cardiomyocytes treated with 2 and 4 mM GA markedly decreased to 13 and 0%, respectively. The cell viability of cardiomyocytes treated with 4 mM GA for 0, 3, 6, 12, and 24 h was assessed using the WST-8 assay. Cell viability decreased in a time-dependent manner (Fig. 1d). Cell viability decreased to 64 and 39% in cardiomyocytes incubated for 3 and 6 h and to 7 and 0% in those incubated for 12 and 24 h, respectively.

### Quantity of intracellular TAGE in cardiomyocytes treated with GA

We performed a slot blot (SB) analysis to investigate the generation of intracellular TAGE in cardiomyocytes treated with 0, 1, 2, and 4 mM GA for 24 h. Intracellular TAGE increased in a dose-dependent manner (Fig. 1e). GA concentrations of 2 and 4 mM significantly increased intracellular TAGE to 28.7 and 38.5 μg/mg protein, respectively. The quantity of intracellular TAGE in cardiomyocytes treated with 4 mM GA for 0, 3, 6, 12, and 24 h, which was analyzed using the SB analysis, increased in a time-dependent manner (Fig. 1f). The GA treatment for 6 h generated 12.0 μg/mg protein of intracellular TAGE, while the same treatment for 12 and 24 h increased the generation of TAGE to 34.2 and 36.7 μg/mg protein, respectively.

### Immunohistochemical analysis of intracellular TAGE in cardiomyocytes treated with GA

**Figure 2:**
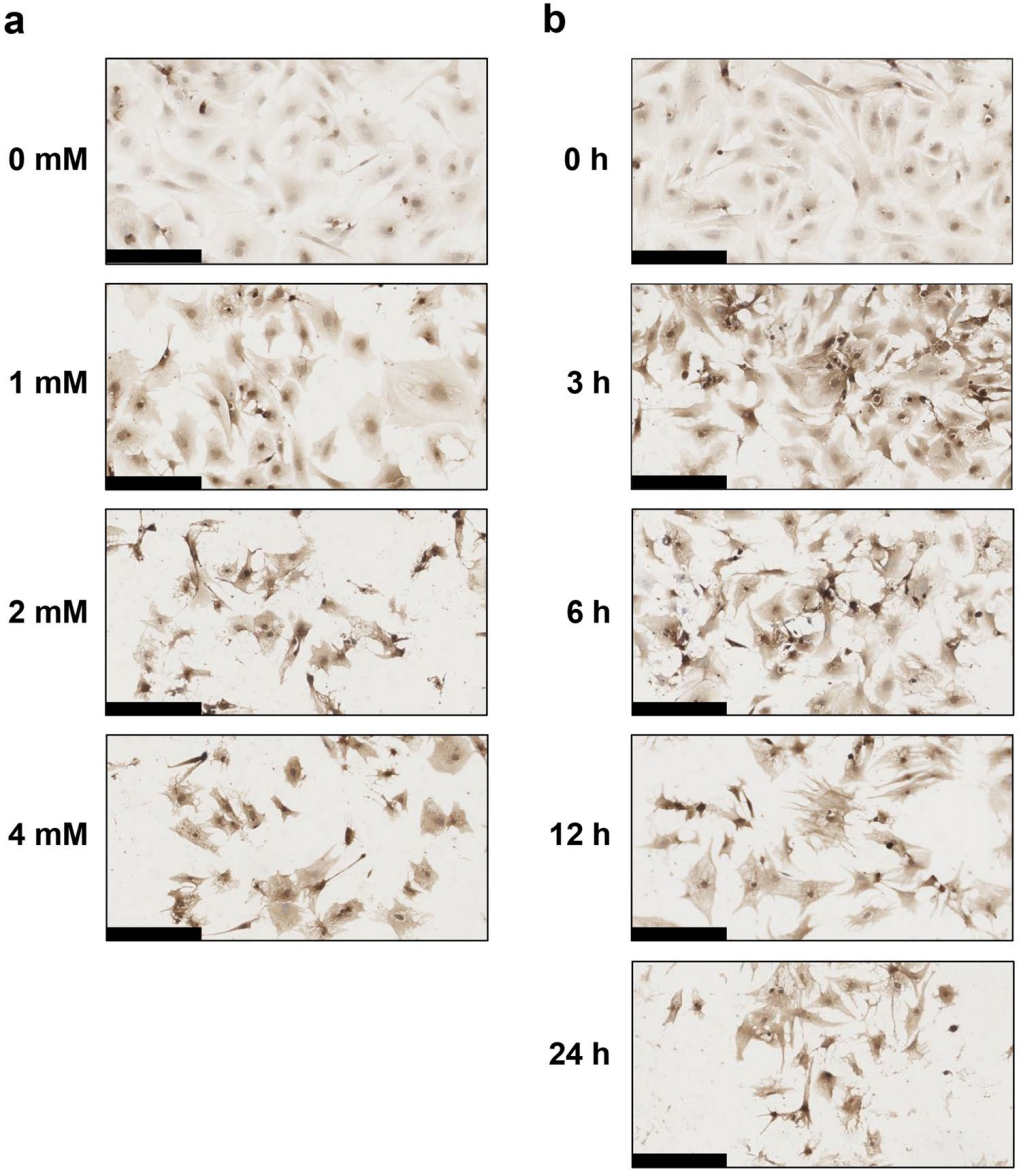
Immunostaining of intracellular TAGE in cardiomyocytes treated with GA. Cardiomyocytes were fixed on the 4-well-type Lab-Tek glass chamber. TAGE-positive areas stained brown in cells. An immunostaining analysis was performed for two independent experiments. The scale bar represents 150 μm.

Intracellular TAGE generation increased in a dose-dependent manner in cardiomyocytes treated with 0, 1, 2, and 4 mM GA for 24 h (Fig. 2a). When cardiomyocytes were treated with 1 mM GA for 24 h, the intensity of the stained areas was stronger than that of the control. Intracellular TAGE in cardiomyocytes treated with 2 and 4 mM GA were strongly stained. We observed areas lacking cells in GA-treated samples. Areas without cells were larger in samples treated with 2 and 4 mM GA than in those treated with 1 mM GA. Intracellular TAGE generation in cardiomyocytes treated with 4 mM GA for 0, 3, 6, 12, and 24 h increased in a time-dependent manner (Fig. 2b). This staining indicated that the generation of intracellular TAGE was greater in cardiomyocytes treated for 3 h with GA than in those treated for 0 h. No significant differences were observed in the staining of intracellular TAGE in cardiomyocytes incubated for 12 and 24 h. Areas without cells expanded in a time-dependent manner.

### LC3-II/LC3-I and p62 in cardiomyocytes treated with GA

**Figure 3:**
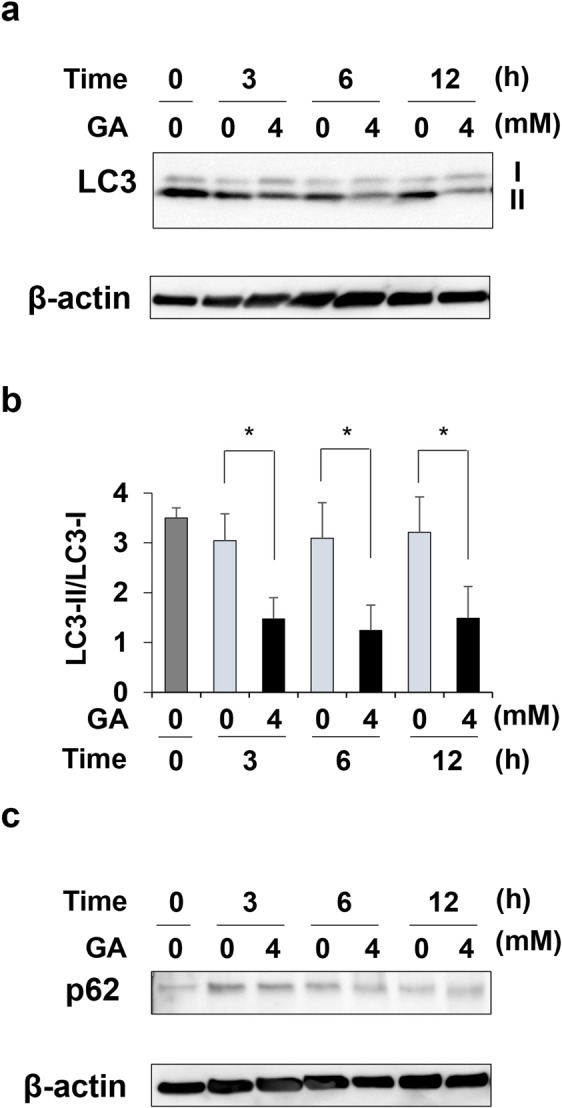
The detection of LC3-I, LC3-II, and p62 with Western blotting and calculation of LC3-II/LC3-I in cardiomyocytes treated with GA. (**a**) The bands of LC3-I and LC3-II were analyzed with Western blotting. The positions of LC3-I and LC3-II are indicated by I and II. Western blotting was performed for three independent experiments. β-actin was used as a loading control. Full-length blots are shown in Supplementary Fig. S1. (**b**) LC3-II/LC3-I was calculated with the band levels of LC3-I and LC3-II. Data are shown as means ± S.D. (N = 3). P-values were based on Tukey’s test. *p < 0.05 vs. the control at each time point. (**c**) The bands of p62 were analyzed with Western blotting. Western blotting was performed in three independent experiments. β-actin was used as the loading control. Full-length blots are shown in Supplementary Fig. S2.

LC3-I, LC3-II, and p62 in cardiomyocytes treated with 4 mM GA for 0, 3, 6, and 12 h were detected using a Western blot analysis, and LC3-II/LC3-I was calculated based on LC3-I and LC3-II levels (Fig. 3). Although LC3-II/LC3-I in cardiomyocytes for controls at 3, 6, and 12 h were approximately 3.0, and LC3-II/LC3-I in cardiomyocytes treated with GA decreased to less than 1.5 (Fig. 3a,b). On the other hand, no changes were observed in p62 in cardiomyocytes treated with 0–4 mM GA at each time point (Fig. 3c).

## Discussion

We previously reported that the activation of the extracellular TAGE receptor for AGEs (RAGE) axis resulted in the generation of intracellular reactive oxygen species and the subsequent activation of nuclear factor-κB in vascular wall cells, which may promote the expression of various atherosclerosis- and inflammation-related genes, thereby contributing to the onset and/or progression of CVD in DM^1^. Our findings indicated that serum TAGE levels positively correlated with risk factors for CVD, including plasminogen activator inhibitor-1^21^, fibrinogen^21^, vascular inflammation^22^, endothelial progenitor cells^23^, plaque progression^24^, the mean amplitude of glycemic excursions^25^, asymmetric dimethylarginine^26^, trimethylamine^27^, and the cardio-ankle vascular index^28^, not only in patients with LSRD, but also in healthy humans. In these studies, the mechanisms of extracellular TAGE or the above risk factors promoting CVD involve vascular disorders. In contrast, we herein revealed that intracellular TAGE were generated in cardiomyocytes. We treated cardiomyocytes with GA to rapidly generate intracellular TAGE, because GA, which is a precursor of TAGE, is generated in cardiomyocytes by three pathways^1,13,14^. (1) Glucose is metabolized to GA *via* glycolysis. (2) Fructose is metabolized to GA *via* the pathway involving fructokinase and aldolase B (fructolysis). (3) Glucose is metabolized to fructose *via* the sorbitol pathway, which regulates aldose reductase and sorbitol dehydrogenase, and this fructose is metabolized to GA *via* fructolysis.

In the present study, cardiomyocytes were treated with GA at a physiological concentration to generate TAGE within 24 h. Taniguchi *et al*. previously demonstrated that islets of the pancreas exposed to 20 mM glucose accumulated 0.025 pmol/islet GA, whereas exposure to 10 mM GA caused the accumulation of 0.12 pmol/islet GA^40^. Based on these findings, Takahashi *et al*. used 2 mM GA in their experiments, which is a similar concentration to that using 20 mM glucose^41^. However, two studies on DM model rats raised for several weeks revealed increases in non-fasting plasma glucose levels by >20 mM^42,43^. The plasma glucose levels of diabetic ketoacidosis patients were previously reported to be 89.7 ± 40.1 mM^44^. We considered the 1, 2 and 4 mM GA treatments *in vitro* to reflect physiological conditions. Cardiomyocytes were then treated with 1, 2 and 4 mM GA, and beating rates, cell viability, and the generation of TAGE were analyzed. We focused on the effects of TAGE on beating rates because it is the most important and characteristic function of cardiac cells^33–36^. The cell death of cardiomyocytes induces heart failure. However, the dysfunctional beating of cardiomyocytes damages the heart because the reduced beating of cardiomyocytes may cause life-threatening arrhythmias and result in ventricular fibrillation^45^. Therefore, we employed rat primary cardiomyocytes to measure beating in the present study. Since human or other mammalian cell lines of cardiomyocytes do not exhibit beating, they were unsuitable for our purposes. The results obtained in the present study showed that beating rates and cell viability decreased with the generation of intracellular TAGE (Figs 1 and 2). Furthermore, we performed immunohistochemistry and observed that some areas of GA-treated samples were devoid of cells. This result indicated that cell destruction and death had occurred (Fig. 2).

In cardiomyocytes treated with 4 mM GA for 6 h (the generation of TAGE was 12.0 μg/mg protein), beating completely stopped and cell viability was 39% (Fig. 1b,d,f). These results indicate that the cessation of beating was induced not only in dead cells, but also in living cells. Intracellular TAGE in cardiomyocytes may decrease beating, and ultimately induce cell death. However, it currently remains unclear whether these mechanisms involve the same or different pathways. To demonstrate that the generation of TAGE decreased beating and cell viability, cardiomyocytes were pretreated with 16 mM aminoguanidine (AG), an inhibitor of the generation of TAGE, for 2 h followed by 2 mM GA for 24 h. AG inhibited decreases in beating and cell viability as well as the generation of TAGE (Supplementary Fig. S3).

When occlusion in vascular disorders, such as that of the coronary arteries, occurs, a sudden decrease in the supply of nutrients and oxygen to heart muscles damages and/or causes the death of cardiomyocytes and impairs cardiac performance if blood flow is not quickly restored^46^. The present results revealed that intracellular TAGE in cardiomyocytes, which may be generated by an excessive intake of glucose and/or fructose, decreased beating rates and induced cell death. These effects may cause life-threatening arrhythmia and potentially result in death *in vivo*^45^.

Moreover, the present results suggest that intracellular TAGE suppress macroautophagy (hereafter referred to as autophagy). Autophagy is an intracellular degradation process that contributes to the recycling of long-lived, aggregated, or misfolded proteins or even entire organelles^37–39^. During this process, the cargo to be degraded becomes enveloped within double-membrane vesicles called autophagosomes, which then fuse with lysosomes for degradation^37–39^. The dysregulation of autophagy has been implicated in several diseases, including cardiomyopathy, cancer, and neurodegeneration^47^. Therefore, we predicted that cardiomyocytes promote autophagy for the degradation of intracellular TAGE as cytotoxic proteins in order to survive. We analyzed LC3-II/LC3-I and p62, which are factors and/or an index of autophagy^38,39^. LC3-II/LC3-I increased, while p62 decreased when the formation of autophagosomes was promoted^38^. Although intracellular TAGE were clearly shown to be cytotoxic proteins in the cell lines of some organs^16,29,31,32^, our results revealed that LC3-II/LC3-I in cardiomyocytes, which generated intracellular TAGE when treated with 4 mM GA for 3, 6, and 12 h, decreased to less than 1.5, whereas LC3-II/LC3-I in the controls at each time point was approximately 3.0 (Figs 1f, 2b and 3a,b). LC3-II/LC3-I did not decrease in a time-dependent manner. Autophagy maintains cellular homeostasis and occurs at basal levels without the degradation of cytotoxic proteins^39,47^. In the present study, TAGE appeared to inhibit the degradation of these proteins. However, functions other than toxic protein degradation might be maintained. To demonstrate that the generation of TAGE decreased LC3-II/LC3-I, we analyzed LC3-II/LC3-I in cardiomyocytes treated with 16 mM AG for 2 h followed by 2 mM GA for 24 h. LC3-II/LC3-I in cardiomyocytes treated with 2 mM GA for 24 h decreased, similar to those treated with 4 mM GA for 3, 6, and 12 h. AG inhibited the decrease in LC3-II/LC3-I in cardiomyocytes (Supplementary Fig. S4).

Since the present results only indicated the suppression of autophagy by a decrease in LC3-II/LC3-I, the inhibition of autophagy needs to be analyzed in further experiments^37,38^ in future studies. The mechanisms responsible for intracellular TAGE reducing LC3-II/LC3-I and the amount of p62 remaining unchanged may be revealed. If the suppression of autophagy by intracellular TAGE is proven, this phenomenon will be very important. Although intracellular TAGE need to be the targets of degradation *via* autophagy, they may actually suppress autophagy. We suggest that the repeated generation of intracellular TAGE and suppression of autophagy markedly damage cardiomyocytes.

Intracellular TAGE may inhibit beating *via* the suppression of autophagy. A previous study reported that LC3-II/LC3-I increased when the beating of cardiomyocytes subjected to anoxia and reoxygenation as an ischemia model recovered to normal levels^48^. In contrast, LC3-II/LC3-I in cardiomyocytes, which generated intracellular TAGE, decreased when beating rates were reduced or completely stopped (Figs 1b,f, 2b and 3a,b). Cell death may be induced *via* the suppression of autophagy because cardiomyocytes do not have the ability to degrade TAGE as cytotoxic proteins.

In conclusion, the present results suggest that intracellular TAGE are generated in cardiomyocytes, decrease beating rates, and induce cell death. They may inhibit autophagy, the role of which is to degrade them, and the cessation of beating and induction of cell death may be caused by the suppression of autophagy. Intracellular TAGE may be generated in human cardiomyocytes as well as rat cardiomyocytes, and directly damage them, resulting in CVD. Future studies are needed to identify TAGE-modified proteins and elucidate the mechanisms underlying the development of CVD by the generation of TAGE.

## Materials and Methods

### Animals

Neonatal Wistar/ST rats (1day old) werse obtained from Sankyo Lab Service Co., Inc. (Tokyo, Japan). All experiments using rats were approved by the Committee on Experimental Animals at Kanazawa Medical University and conducted in accordance with their guidelines.

### Reagents and Antibodies

Dulbecco’s modified Eagle’s medium and Ham’s F12 Nutrient Mixtures s(D-MEM and HAM-F12 (1:1)), penicillin-streptomycin solution, and bromodeoxyuridine (BrdU) were obtained from Sigma-Aldrich (MO, USA). Fetal bovine serum (FBS) was purchased from Bovogen-Biologicals (VIC, Australia). GA was purchased from Nacalai Tesque, Inc. (Kyoto, Japan). The WST-8 assay kit, 3-[(3-cholamido-propyl)-dimethyl-ammonio]-1-propane sulfonate) (CHAPS), and 3,3′-diaminoenzidine tetrahydrochloride (DAB) were obtained from Dojindo Laboratories (Kumamoto, Japan). Ethylenediamine-N,N,N′,N′-tetraacetic acid (EDTA)-free protease inhibitor cocktail was obtained from Roche Applied Science (Penzberg, Germany). The protein assay kit for the BCA method was purchased from Thermo Fisher Scientific Inc. (MA, USA). The protein assay kit for the Bradford method was obtained from Takara Bio, Inc. (Otsu, Japan). The Western re-probe kit was purchased from Funakoshi Co., Ltd. (Tokyo, Japan). A horseradish peroxidase (HRP)-linked molecular marker was obtained from Bionexus (CA, USA). Anti-LC3 and anti-p62 antibodies were purchased from Cell Signaling Technology Japan K. K. (Tokyo, Japan), and an anti-β-actin antibody was obtained from Abcam (Cambridge, UK). A HRP-linked goat anti-rabbit IgG antibody was purchased from DAKO (Glostrup, Denmark) and HRP-linked goat anti-mouse and HRP-linked donkey anti-rabbit IgG antibodies were obtained from Thermo Fisher Scientific Inc. All other reagents and kits not indicated were purchased from Wako Pure Chemical Industries, Ltd. (Osaka Japan). GA-derived AGE-bovine serum albumin (TAGE-BSA) and an anti-TAGE antibody were prepared as described priviously^49^.

### Cell culture of cardiomyocytes

The ventricles of neonatal Wistar/ST rats (1day old) were used. Small pieces of the ventricles were incubated in 0.05% trypsin solution at 37 °C for 5 min and the supernatant was removed. A gel of small ventricle pieces was incubated in 0.1% trypsin solution and digested by shaking at 37 °C for 10 min. These procedures were performed four or five times. Thereafter, the cell suspension collected was centrifuged at 300 × *g* for 5 min. Cell suspensions were filtered using a 40-μm filter. The cells that were filtered were seeded on a 100-mm dish and incubated for 1 h in a CO_2_ incubator. The cell suspension containing unattached cells was centrifuged at 300 × *g* for 10 min. It was then diluted and inoculated at an appropriate cell density (3.0 × 10^5^ cells/ml) into 48-well microplates, 60-mm dishes, glass coverslips in 35-mm dishes, and 4-well-type Lab-Tek glass chambers (Nalge Nunc, New York, USA). Cells were cultured in D-MEM and HAM-F12 (1:1) including 10% FBS, 100 U/ml penicillin, and 100 μg/ml streptomycin with 0.1 mM BrdU for 48 h. Cells were changed to medium without BrdU and prepared for examination. Cells were incubated with various concentrations of GA (0, 1, 2, and 4 mM) for 0–24 h.

### Beating rate assay of cardiomyocytes

The beating rates of cardiomyocytes were investigated as described previously^50,51^ with some modifications. Briefly, culture plates were transferred to the incubator (37 °C, 5% CO_2_) of an inverted microscope (Nikon Co., Tokyo, Japan), and 4 small labeled circular areas (diameter of 2 mm) in each 35-mm dish were inspected. The results obtained were recorded with a DVD recorder (Victor Co., Osaka, Japan) and selected cells were counted for 30 sec each time.

### Cell viability of cardiomyocytes

Cell viability was assessed using the WST-8 assay. Twenty microliters of WST-8 reagent was added to 48-well microplates in which cardiomyocytes were cultured in medium (250 μl), and then incubated at 37 °C for 3 h in a CO_2_ incubator. Thereafter, 100 μl of the supernatant fluid was transferred to 96-well microplates. Absorbance was measured at 450 and 655 nm using a microplate reader (Bio-Rad, CA, USA). Background absorbance was measured in medium without cells and subtracted from experimental values.

### SB analysis

This analysis was performed as previously described with some modifications^32^. Cells were washed with phosphate-buffered saline (PBS)(-) without Ca and Mg, and lysed in buffer [a solution of 2 M thiourea, 7 M urea, 4% CHAPS, and 30 mM Tris, and a solution of EDTA-free protease inhibitor cocktail (9:1)]. Cell extracts were then incubated on ice for 20 min, centrifuged at 10,000 × *g* at 4 °C for 15 min, and the supernatant was collected as cell extracts. Protein concentrations were measured by the protein assay kit for the Bradford method using BSA as a standard. In the detection of TAGE, equal amounts of cell extracts, the HRP-linked molecular marker, and TAGE-BSA were loaded onto polyvinylidene difluoride (PVDF) membranes (0.45 μm; Millipore, MA, USA) fixed in the SB apparatus (Bio-Rad). PVDF membranes were then cut to prepare two membranes, and were blocked at room temperature for 1 h using 5% skimmed milk in PBS containing 0.05% Tween 20 (SM-PBS-T). After this step, we used 0.5% of SM-PBS-T for washing or as the solvent of antibodies. After washing twice, membranes were incubated with (1) the anti-TAGE-antibody (1:1,000) or (2) neutralized anti-TAGE-antibody (a mixture of the anti-TAGE-antibody (1:1,000) and 250 µg/ml of TAGE-BSA) at 4 °C overnight. Membranes were then washed four times. Proteins on the membrane were incubated with the HRP-linked goat anti-rabbit IgG antibody (1:2,000) at room temperature for 1 h. After washing three times with PBS-T, membranes were moved into PBS. Immunoreactive proteins were detected with the ImmunoStar LD kit and band densities on the membranes were measured using the LAS-4000 fluorescence imager (GE Healthcare, Tokyo, Japan). The densities of HRP-linked molecular marker bands were used to correct for differences in densities between membranes. The amount of TAGE in cell extracts was calculated based on a calibration curve for TAGE-BSA.

### Immunostaining of TAGE in cardiomyocytes

Cells in 4-well-type Lab-Tek glass chambers were incubated in 4% paraformaldehyde at room temperature for 20 min, rinsed with PBS, permeated with 0.1% Triton X-100 for 10 min, and then rinsed PBS, 0.1% BSA-PBS(−) (BSA-PBS) and incubated with 3% BSA-PBS for blocking for 1 h. After being washed with 0.1% BSA-PBS, cultured cells were incubated with the anti-TAGE antibody, which was described previously, and dissolved in 1% BSA-PBS at a dilution of 1:100 for 1 h. Cells were then washed three times with 0.1% BSA-PBS and incubated with the HRP-linked goat anti-rabbit IgG antibody (1:100) for 1 h. After washing with 0.1% BSA-PBS (three times) and PBS, cells were incubated with 0.05% DAB and 0.015% H_2_O_2_ in PBS for 5 min. Thereafter, cells were counterstained briefly with hematoxylin, and observed under the NanoZoomer slide scanner (Hamamatsu Photonics K. K., Hamamatsu, Japan).

### Western blot analysis

Cells on dishes were washed with PBS(−) two times and lysed in buffer [radioimmunoprecipitation assay (RIPA) buffer (Thermo Fisher Scientific Inc.) and a solution of EDTA-free protease inhibitor cocktail (9:1)]. Protein concentrations were assessed by the protein assay kit for the BCA method using BSA as a standard. Cell extracts were mixed with sodium dodecyl sulphate (SDS) sample buffer (Bio-Rad) and 2-mercaptoethanol (Sigma-Aldrich), and then heated at 95 °C for 5 min. Equal amounts of cell extracts were resolved by 4–15% gradient SDS-polyacrylamide gel electrophoresis and transferred onto PVDF membranes. The membranes were blocked at room temperature for 30 min using 5% SM-PBS-T. After this step, we used 0.5% SM-PBS-T for washing or as the solvent of antibodies. After washing twice, the membranes were incubated with the anti-LC3 antibody (1:1,000) or anti-p62 antibody (1:1,000). The membranes were washed four times and incubated with the HRP-linked goat anti-mouse IgG antibody (1:5,000) or HRP-linked donkey anti-rabbit IgG antibody (1:2,000). Washing and the detection of immunoreactive proteins were performed followed by a SB analysis. Equivalent sample loading was confirmed by stripping membranes with the Western re-probe kit, and this was followed by blotting with anti-β-actin (1:1,000).

### Statistical analysis

Stat Flex (ver. 6) software (Artech Co., Ltd.) was used for statistical analyses. Data are expressed as means ± S.D. When statistical analyses were performed on data, significant differences in the means of each group were assessed by a one-way Analysis of Variance (ANOVA). We then used Dunnett’s test and Tukey’s test for an analysis of variance. P-values < 0.05 were considered to be significant.

## Supplementary information

Supplementary Information
