# Supplementary material for: Intracellular toxic advanced glycation end-products in cardiomyocytes may cause cardiovascular disease
Source: Sci Rep. 2019 Feb 14;9:2121. doi: 10.1038/s41598-019-39202-5 (PMC6375929; doi:10.1038/s41598-019-39202-5)

Supplementary Information

Intracellular toxic advanced glycation end-products in cardiomyocytes may cause cardiovascular disease

Takanobu Takata*, Akiko Sakasai-Sakai, Tadashi Ueda, & Masayoshi Takeuchi

Department of Advanced Medicine, Medical Research Institute, Kanazawa Medical University, Uchinada-machi, Ishikawa 920-0293, Japan

*Correspondence and requests should be addressed to Takanobu Takata, PhD (E-mail: takajjjj@kanazawa-med.ac.jp).

Supplementary Materials and Methods

Reagent

Aminoguanidine was purchased from Wako Pure Chemical Industries, Ltd. (Osaka Japan).

Methods

Cell culture of cardiomyocytes. The method of cell culture before the treatment with AG was the same as that described in the "Materials and Methods". Cells were pretreated with 16 mM AG for 2 h followed by 2 mM GA for 24 h.

Beating rate assay of cardiomyocytes. The beating rates of cardiomyocytes were investigated as described previously[50,51] with some modifications. Briefly, culture plates were transferred to the incubator (37°C, 5% CO_2, Imug-2-KIW, TOKAI HIT Co., Ltd. Fujinomiya, Japan) of an inverted microscope (BZ-X710, Keyence Co., Osaka, Japan), and 4 areas that were randomly selected in each 35-mm dish were inspected. Videos were recorded with a personal computer. The beating of cells was counted for 30 sec each time.

Supplementary Figure legends

Supplementary Figure S1.

Full-length blot of Figure 3a. (**a**) The LC3-I and LC3-II bands were detected on polyvinylidene difluoride (PVDF) membranes. The open box replaces the LC3-I and LC3-II bands indicated in Figure 3a. The positions of LC3-I and LC3-II are indicated by I and II. (**b**) The β-actin band was detected on the PVDF membrane. The open box replaces the β-actin band indicated in Figure 3a.

Supplementary Figure S2.

Full-length blot of Figure 3c. (**a**) The p62 band was detected on the PVDF membrane with high sensitivity and an exposure time of 5.0 sec. The open box replaces the p62 band indicated in Figure 3c. (**b**) The p62 band was detected on the PVDF membrane with standard sensitivity and an exposure time of 2.0 sec. The p62 band is located in the open box. (**c**) The p62 band was detected on the PVDF membrane with standard sensitivity and an exposure time of 20 sec. The p62 band is located in the open box. (**d**) The β-actin band was detected on the PVDF membrane. The open box replaces the β-actin band indicated in Figure 3c.

Supplementary Figure S3.

The beating rate, cell viability, and quantity of intracellular TAGE in cardiomyocytes treated with 0 or 16 mM AG for 2 h followed by 0 or 2 mM GA for 24 h. (**a**) Beating rates were assessed in three independent experiments. One experiment was performed to count the beating rates of cardiomyocytes in 4 areas that were randomly selected in 35-mm dishes in order to calculate the average. Data are shown as means ± S.D. (N=3). P-values were based on Tukey's test. $^{**}p<0.01$ vs. the control. $^{\#\#}p<0.01$ vs. the treatment with 2 mM GA. (**b**) Cell viability was assessed using the WST-8 assay. This assay was performed in three independent experiments. One experiment was performed using 4 wells to calculate the average. Data are shown as means ± S.D. (N=3). P-values

were based on Tukey's test. ***p*<0.01 vs. the control. ##*p*<0.01 vs. the treatment with 2 mM GA. (**c**) Intracellular TAGE were analyzed with a slot blot (SB) analysis. Cell lysates (2.0 μg of protein/lane) were blotted onto a polyvinylidene difluoride (PVDF) membrane. The amount of TAGE was calculated based on a calibration curve for GA-derived AGE-BSA (TAGE-BSA). A SB analysis was performed in three independent experiments. Data are shown as means ± S.D. (N=3). P-values were based on Tukey's test. ***p*<0.01 vs. the control. ##*p*<0.01 vs. the treatment with 2 mM GA.

Supplementary Figure S4.

The detection of LC3-I and LC3-II with Western blotting (WB) and calculation of LC3-II/LC3-I in cardiomyocytes treated with 0 or 16 mM AG for 2 h followed by 0 or 2 mM GA for 24 h. (**a**) The bands of LC3-I and LC3-II were analyzed with WB. The positions of LC3-I and LC3-II are indicated by I and II. WB was performed for three independent experiments. β-actin was used as a loading control. (**b**) LC3-II/LC3-I was calculated with the band levels of LC3-I and LC3-II. Data are shown as means ± S.D. (N=3). P-values were based on Tukey's test. ***p*<0.01 vs. the control. ##*p*<0.01 vs. the treatment with 2 mM GA.

Supplementary Figure S1

a

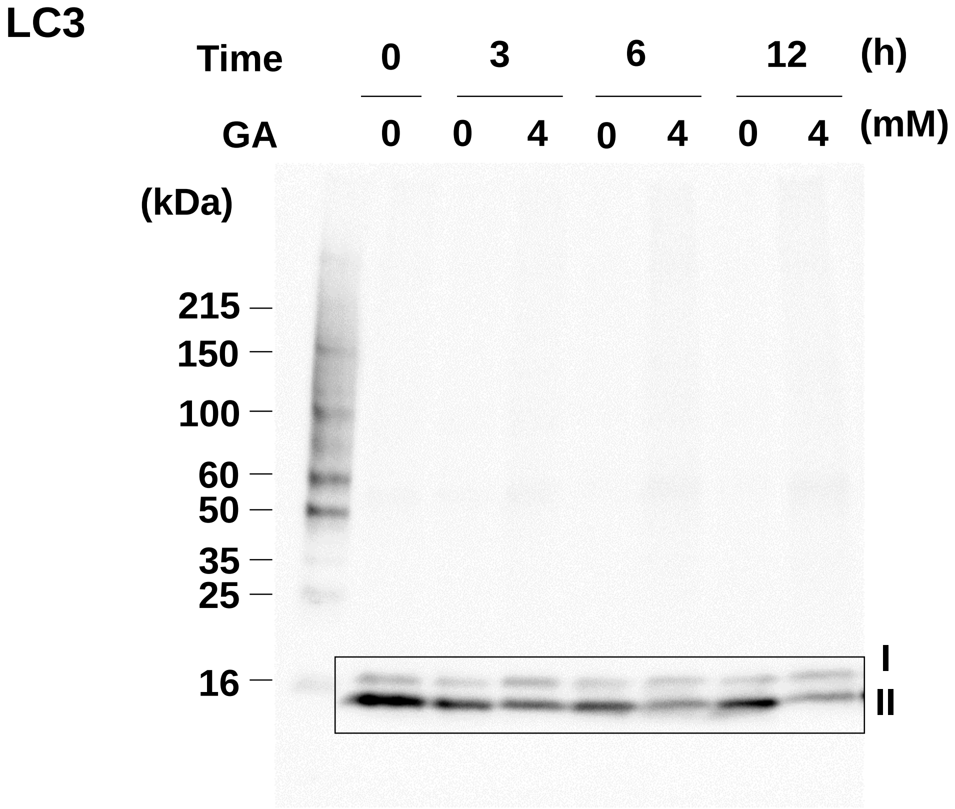

b

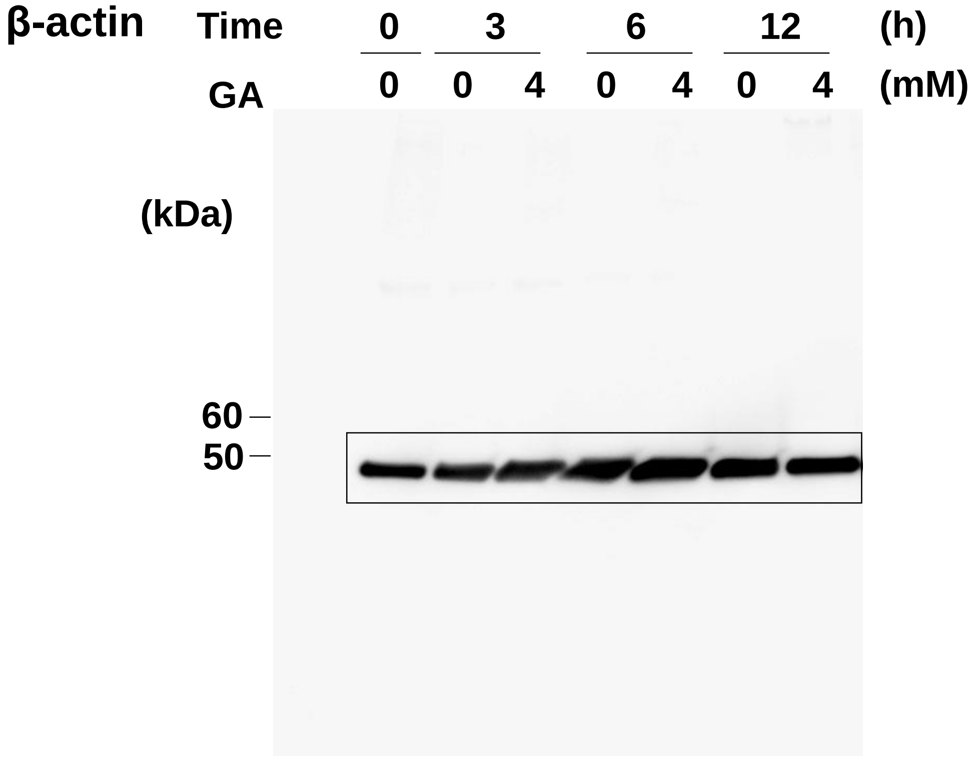

Supplementary Figure S2

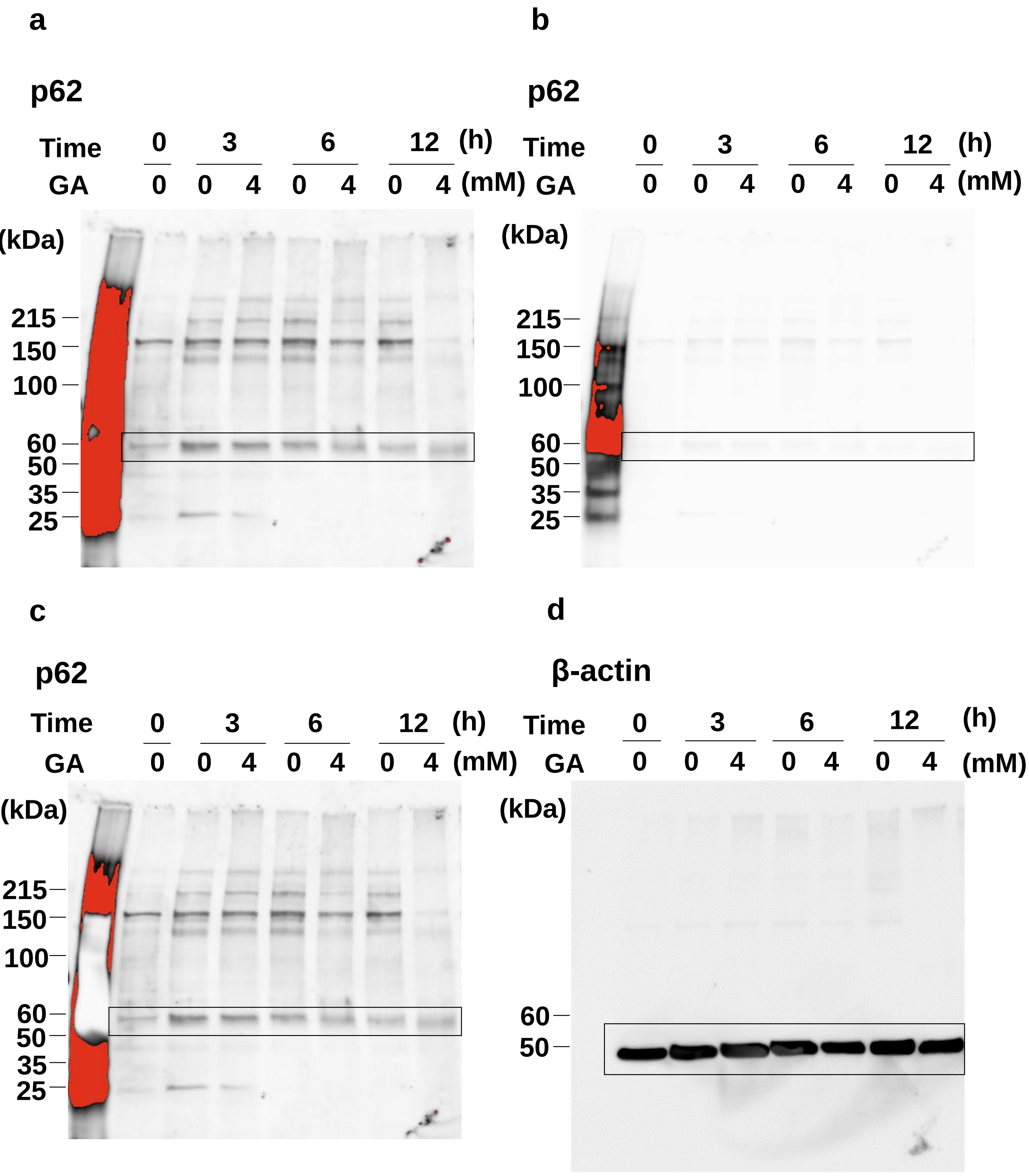

Supplementary Figure S3

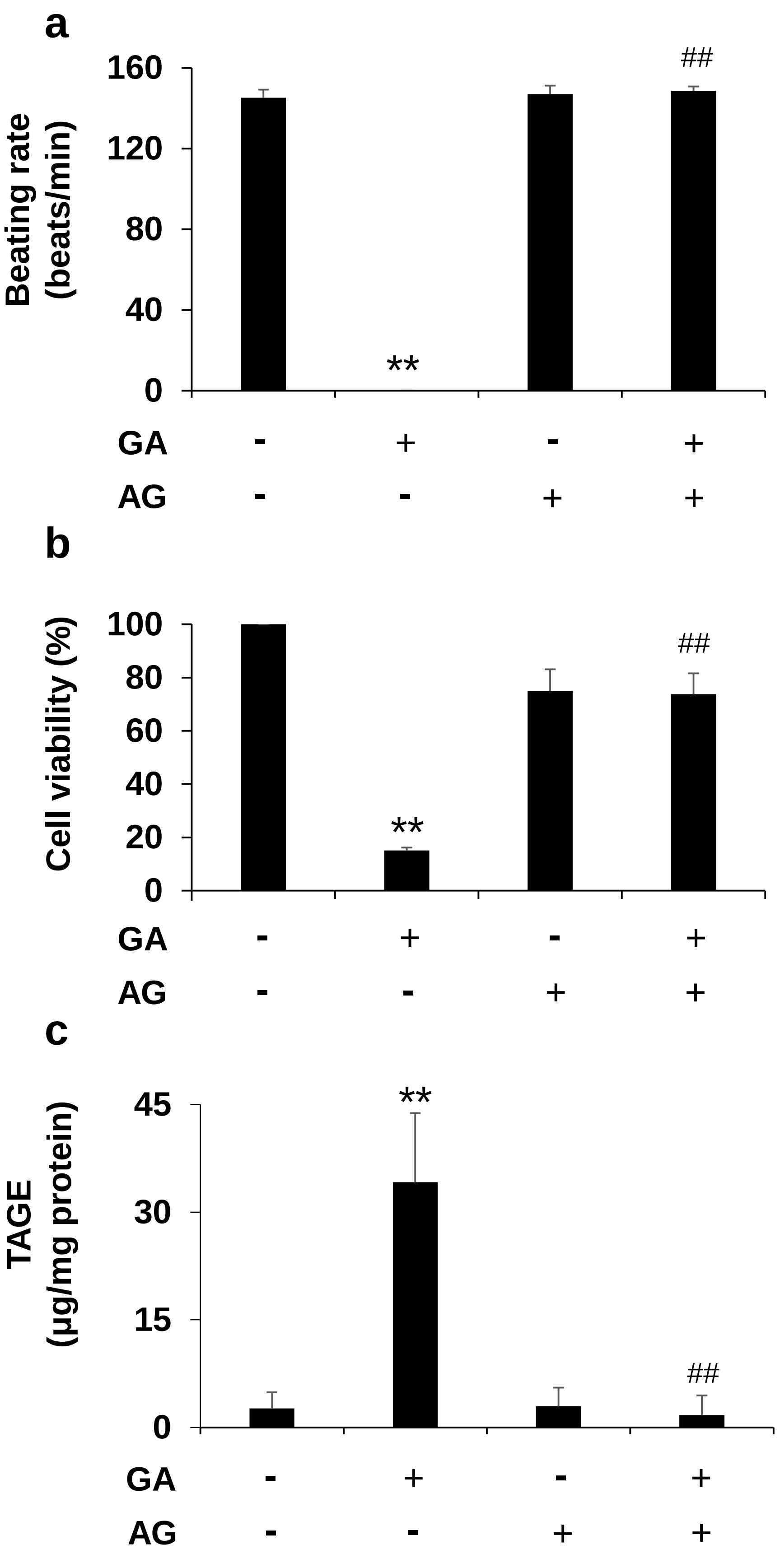

Supplementary Figure S4

a

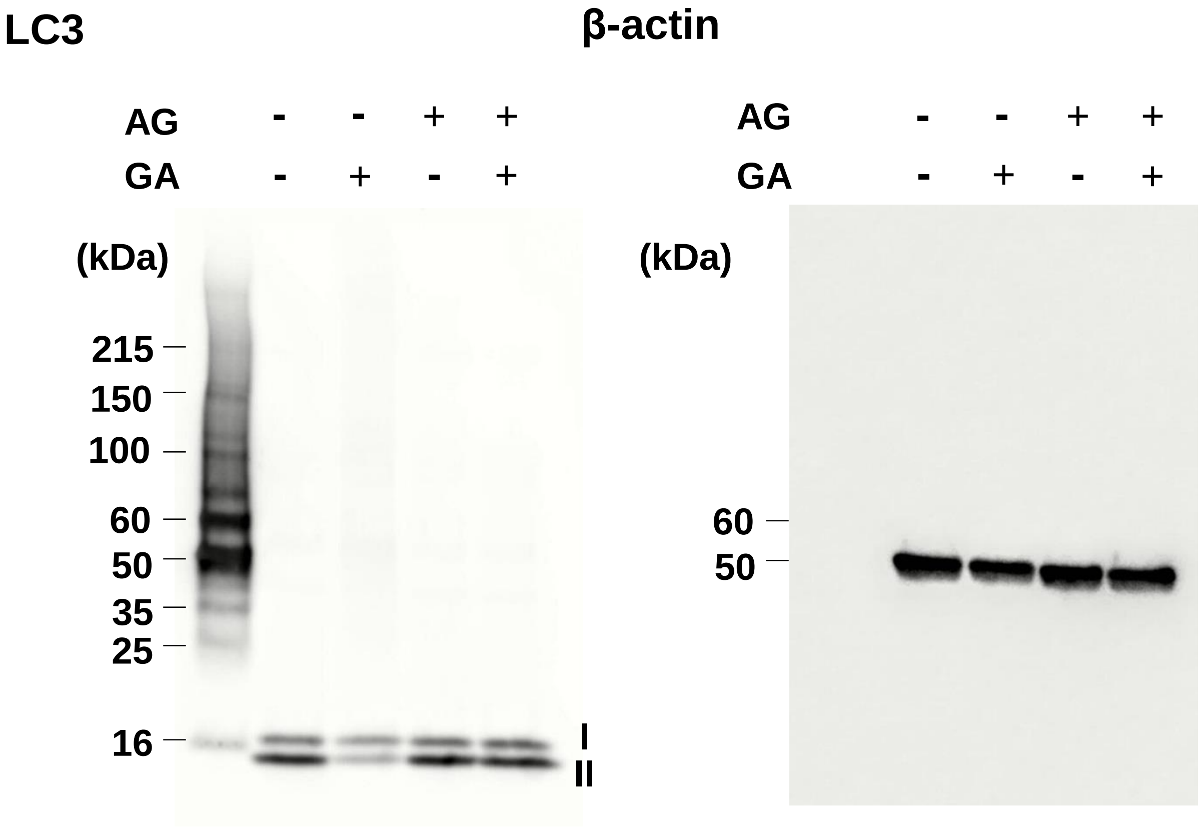

b

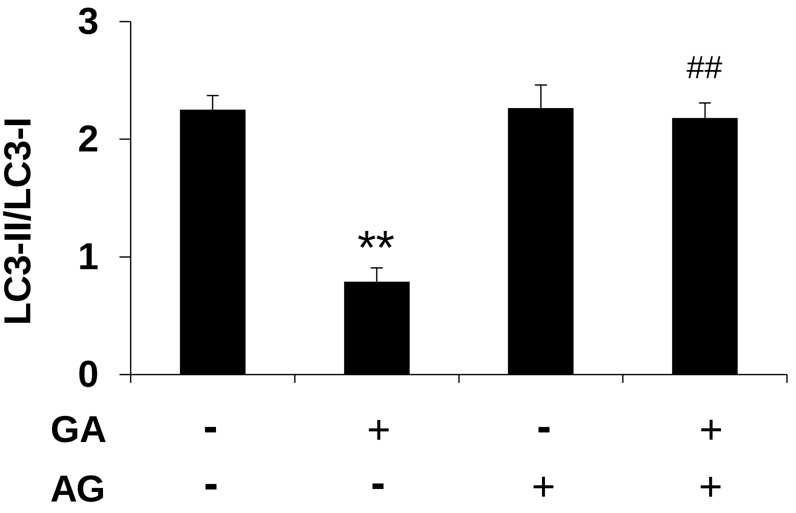

Supplement: Supplementary file 1 — Supplementary Information [file 41598_2019_39202_MOESM1_ESM.pdf]
